# Use of Thromboelastography in Coronary Artery Bypass Grafting in a Patient With Factor Ⅴ Deficiency With Platelet Function Disorders: A Case Report and Literature Review

**DOI:** 10.7759/cureus.58185

**Published:** 2024-04-13

**Authors:** Shino Kawazoe, Takahiro Tamura, Takehito Sato, Akari Matsuura, Kimitoshi Nishiwaki

**Affiliations:** 1 Department of Anesthesiology, Nagoya University Hospital, Nagoya, JPN; 2 Department of Anesthesiology, Nagoya University Graduate School of Medicine, Nagoya, JPN

**Keywords:** cardiopulmonary bypass, platelet function disorders, coronary artery bypass graft, thromboelastography, factor ⅴ deficiency

## Abstract

Reports on cases of factor Ⅴ (FⅤ) deficiency complicated by platelet function disorders in patients undergoing cardiac surgery are rare, and the utilization of thromboelastography in such cases is limited. This case presents a unique case of FⅤ deficiency complicated by platelet function disorders, highlighting the significance of tailored transfusion strategies guided by thromboelastography (TEG).

A 64-year-old hemodialysis patient who was diagnosed with FⅤ deficiency 24 years prior presented for an on-pump coronary artery bypass graft. The decrease in FⅤ activity on preoperative examination was mild. Based on this finding, it was determined that preoperative fresh frozen plasma supplementation was not required. However, the case was complicated by platelet function disorders; therefore, a preoperative transfusion of platelet concentrate was performed to correct the decreased platelet function, enabling subsequent surgery. Intraoperative and postoperative transfusion strategies were guided by TEG.

This study highlights TEG-guided transfusion management as a viable option for patients with FⅤ deficiency complicated by platelet function disorders.

## Introduction

Factor Ⅴ (FⅤ) deficiency is an extremely rare hemorrhagic disease that can be classified as congenital or acquired [1]. Most patients with this disorder present with mild to moderate bleeding symptoms and rarely require transfusion of blood products or hemostatic intervention [1]. However, under highly invasive circumstances such as trauma or surgical procedures (including tooth extraction), even patients with mild bleeding symptoms may be susceptible to abnormal bleeding [2,3], and FⅤ activity should be maintained above 15%-25% in the perioperative period [4,5]. In Japan, where recombinant FⅤ products are currently unavailable, fresh frozen plasma (FFP) has been used when supplementation is required [4]. Cardiac surgery in patients with FⅤ deficiency has been rarely reported, with even rarer instances involving cases of this condition being complicated by platelet function disorders. This study reports the case of a patient with FⅤ deficiency complicated by platelet function disorders who underwent an on-pump coronary artery bypass graft (CABG).

This article was previously presented as a meeting abstract at the 27th Annual Meeting of the Japanese Society of Cardiovascular Anesthesiologists on September 17, 2022.

## Case presentation

A 64-year-old man with a past medical history of end-stage kidney disease who was hemodialysis dependent was diagnosed with FⅤ deficiency 24 years ago based on the results of a panel of blood tests performed by his previous physician during a renal biopsy. The blood tests revealed prolonged prothrombin time (PT) and activated partial thromboplastin time (APTT). The patient had been on maintenance hemodialysis for 20 years and had no history of any special preoperative procedures or bleeding symptoms during the perioperative period of previous peritoneal dialysis catheter placement, catheter removal, or forearm arteriovenous shunt creation. However, the patient experienced prolonged bleeding, requiring the transfusion of eight units of FFP, while tooth extraction was performed one year ago. 

The patient presented with chest pain and shortness of breath on exertion and underwent coronary computed tomography angiography, revealing triple vessel disease. The patient was scheduled to undergo on-pump CABG and was referred to our hospital two months ago. Blood tests at the previous hospital revealed a lupus anticoagulant level of 1.07, while the APTT cross-mixing test revealed a coagulation factor-deficient pattern. Preoperative examination performed at our hospital revealed that PT was 13.3 seconds, PT-International Normalized Ratio was 1.27, APTT was 45 seconds, FⅤ activity was 24%, and platelet count was 9.7×104/μL. Platelet aggregation studies were performed using ADP (3.0μM), collagen (2.0μg), and ristocetin (1.5mg), which revealed decreased platelet aggregation function. Compared to samples comparable to the patient's platelet count, aggregation rates were 54.6% for ADP, 0% for collagen, and 62.0% for ristocetin. Therefore, the patient did require preoperative platelet supplementation owing to decreased platelet count and aggregation. Since FⅤ activity was low but maintained at 24%, preoperative FFP supplementation was deemed unnecessary, and 30 units of platelet concentrates (450ml) were transfused at the time of hemodialysis on the day before the surgery.

Although bone marrow aspiration was performed preoperatively in our patient, the test results for myelodysplastic syndromes were negative, and no significant findings were suggestive of other hematological diseases. 

**Figure 1:**
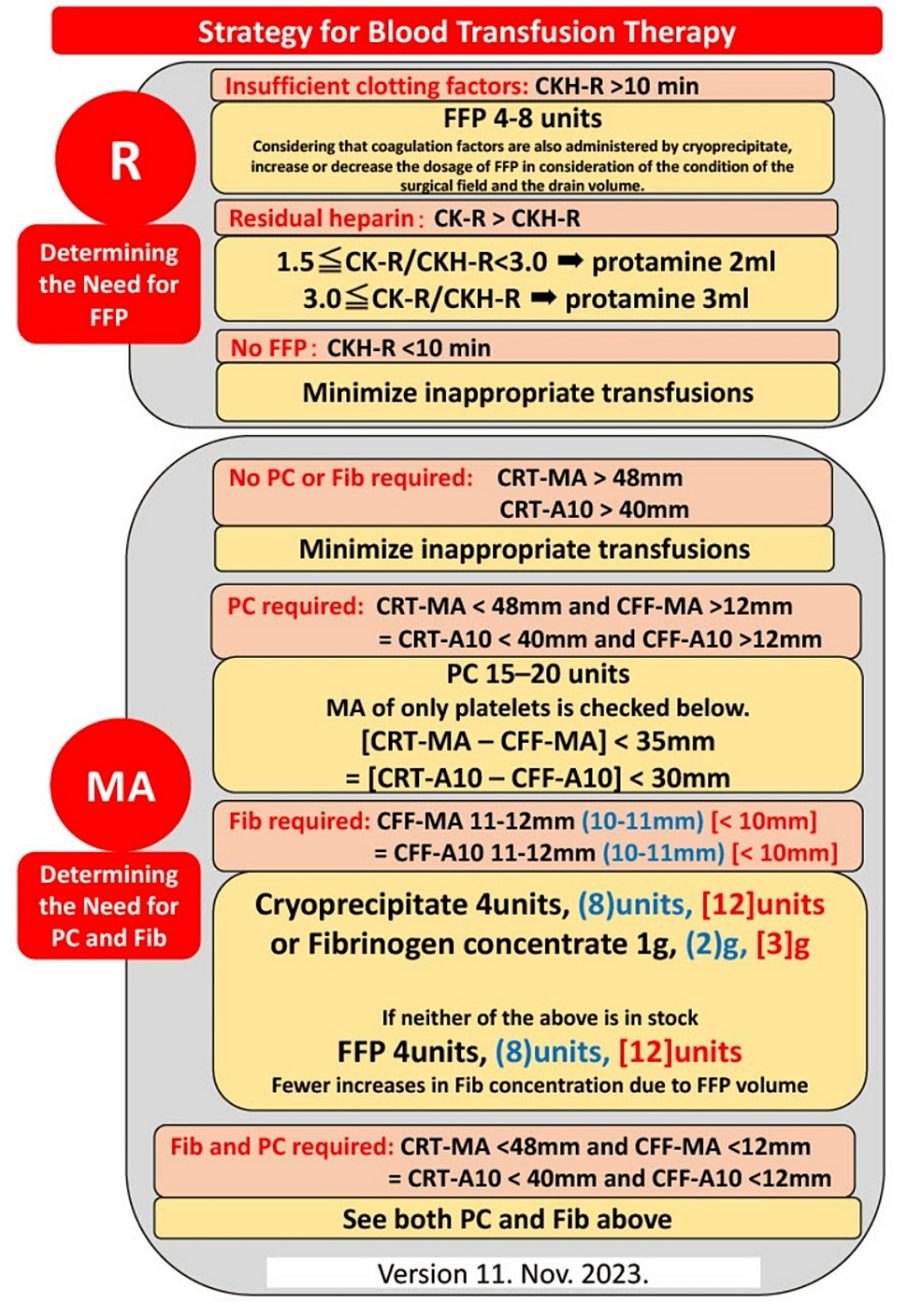
Strategy for blood transfusion therapy Image credits: Takahiro Tamura

Standard monitoring was initiated, and fentanyl and midazolam were administered intravenously to induce general anesthesia. Remifentanil and rocuronium were used to facilitate tracheal intubation. General anesthesia was maintained with air, oxygen, remifentanil, and volatile anesthetics. Heparin sodium (300 U/kg) was administered before initiating cardiopulmonary bypass (CPB), and additional bolus doses of heparin (50 U/kg) were administered to maintain an activated clotting time (ACT, ACT+, Hemochron®, HEIWA BUSSAN CO., LTD., Tokyo, Japan) of at least 400 seconds. After the completion of the surgery, the patient was weaned off CPB. Protamine (3 mg/kg) was administered to reverse heparin. Red blood cell concentrates were transfused to maintain a hemoglobin level over 7-8 g/dL during CPB. The TEG®6s (Hemonetics LLC, Tokyo, Japan) algorithm (Figure 1) is used in operating rooms and intensive care units at our hospital to determine the required transfusion formulation [6].

The fibrinogen level was 229 mg/dL during CPB, and the ACT after the administration of protamine was 110 seconds. Blood samples acquired after protamine administration revealed that the reaction times of citrated kaolin (CK-R) and citrated kaolin with heparinase (CKH-R) were 14.9 seconds and 9.7 seconds, respectively (Table 1), and 4 units of FFP and 12 units of cryoprecipitate were administered after discussion between anesthesiologists and cardiac surgeons about the fact that he was a dialysis patient and that it was safer to administer coagulation factor supplementation than the state of coagulation factors near the cut-off line. We did not administer protamine because the CK-R was just 1.5 times the CKH-R and not significantly different. Platelet replacement was deemed unnecessary as the maximum amplitude of citrated rapid TEG (CRT-MA) was in the normal range and the platelet count was 11.4×104/μL. The total intraoperative blood loss was 1,135 mL.

**Table 1:** Perioperative TEG®6s results *1 After weaning off CPB and administering protamine *2 After admission to the ICU after the surgery *3 POD 1 morning CK-R: reaction time of citrated kaolin, CRT-R: reaction time of citrated rapid TEG, CKH-R: reaction time of citrated kaolin with heparinase, CRT-MA: maximum amplitude of citrated rapid TEG, CFF-MA: maximum amplitude of citrated functional fibrinogen, CPB: cardiopulmonary bypass, ICU: intensive care unit, POD: post operative day

	After CPB^*1^	ICU^*2^	POD 1^*3^
CK-R, min	14.9	6.1	10.5
CRT-R, min	1.2	0.5	1.1
CKH-R, min	9.7	6.2	10.1
CRT-MA, mm	63.7	62.4	65.5
CFF-MA, mm	30.3	26.9	32.4

TEG was performed immediately after admission to the intensive care unit and on the subsequent morning (Table 1). The TEG results acquired immediately after admission were normal, and a blood transfusion was not performed, considering clinical findings such as the amount of drain bleeding. The TEG results acquired on the subsequent morning revealed some deviations (CK-R, 10.5; CKH-R, 10.1; Table 1); however, the amount of drain bleeding was negligible, and blood transfusion was not performed, considering the clinical findings. Postoperative chest tube and mediastinal tube output was 100 mL at 2 hours, and bleeding did not increase after the patient started receiving aspirin on the first postoperative day. The drain was removed on the second postoperative day, with a total bleeding volume of 340 mL. The patient was extubated on the day of the surgery and transferred from the intensive care unit without hemorrhagic complications three days after the surgery. The patient was discharged post-operatively on day 26.

## Discussion

FⅤ deficiency is an extremely rare autosomal recessive genetic disorder, with a reported incidence rate of approximately one in a million [1,7], and can be classified as congenital or acquired FⅤ deficiency. Some coagulation disorders, including factor V deficiency, are suspected if there is a history of bleeding, physical examination findings, or abnormal blood tests on preoperative screening [8]. Acquired FⅤ deficiency has been known to occur due to severe liver injury, disseminated intravascular coagulation, and exposure to topical hemostatic products containing bovine thrombin as a result of autoimmune cross-reactivity against bovine factor V contaminating the product [9]. Since the condition in the present case was not complicated by liver disease or disseminated intravascular coagulation and the finding that the cross-mixing test revealed a coagulation factor-deficient pattern, it was inferred that the patient had congenital FⅤ deficiency. Congenital FⅤ deficiency can cause significant changes in laboratory results during long-term follow-up, necessitating preoperative evaluation, including coagulation system tests for each invasive procedure [10]. The patient again underwent preoperative examination after admission, and although the FⅤ activity level was low at 24%, it was above the 20% threshold for transfusion therapy. The platelet count was low and platelet function had declined; therefore, preoperative platelet transfusion was planned, in addition to using TEG to guide intraoperative transfusion.

The significance of this case report lies in its exploration of blood transfusion management in patients with FⅤ deficiency during the perioperative period of cardiac surgery, employing TEG to guide decisions between plasma or platelet replacement during the perioperative period.

As part of the perioperative management for patients undergoing cardiac surgery using CPB, our hospital uses a protocol for measuring TEG after weaning from CPB and admission to the intensive care unit to determine whether transfusion should be performed to ensure appropriate management [6]. Although the reaction time (R) result was just below the prescribed limit (CKH-R<10 mm) in TEG measurement after protamine administration, the anesthesiologists and cardiac surgeons determined the use of cryoprecipitate considering the total amount as the patient had FⅤ deficiency and was undergoing dialysis. However, based on the TEG results, the plasma products could have been reduced by using 8 units instead of 12 or only 4 units. In addition, administration of platelets during the surgery was not required, as the maximum amplitude (MA) results of TEG were good.

In a literature review, we found many cases in which FFP was used for perioperative transfusion in patients with FⅤ deficiency (Table 2) but repeat administration of FFP resulted in volume overload and consequently caused pulmonary edema in some cases (Table 2: No. 1, 5, and 25). Therefore, the use of cryoprecipitate for coagulation factor replacement in this case may have prevented volume overload. In addition, the use of TEG and transfusion of only the necessary amount of blood in the transfusion strategy may be useful to avoid volume overload. Our search for the use of perioperative TEG in patients with FV deficiency revealed only three cases (Table 2: No. 16, 17, and 29), so our report is significant as a case in which TEG was used to determine the transfusion requirement.

**Table 2:** Transfusion therapy for surgical intervention in patients with FV deficiency in the literature MeSH search formulas are as follows: they were searched on December 1, 2022. ((factor V deficiency [MeSH Terms]) AND (surgery[MeSH Terms])) AND (transfusion[MeSH Terms]) and (cardiac surgery[MeSH Terms]) AND (factor v deficiency[MeSH Terms]). A total of 50 articles were retrieved. There were five articles that were duplicates in two MeSH search formulas, six articles that did not perform surgery, five articles that were over 30 years old and could not be referenced in the text, four articles that were not transfused or lacked details, and one article that did not discuss FⅤ deficiency. As a result, 29 articles were included, and all the literature contents were checked. FFP: fresh frozen plasma, PE: plasma exchange, RBC: red blood cell, CABG: coronary artery bypass grafting, DDAVP: 1-desamino-8-D-arginine vasopressin, rFⅦa: recombinant activated factor Ⅶ, S/DP: solvent detergent-treated plasma, MB FFP: methylene blue-treated FFP, SD FFP: solvent detergent FFP, PC: platelet concentrate, VI FFP: virus-inactivated FFP

Author, year	Surgical procedure	Perioperative transfusion therapy	Use of thromboelastography	Comments
Drzymalski DM, 2019	Cesarean delivery with epidural anesthesia	FFP, Platelets	Not reported	The patient developed pulmonary edema.
Brown L, 2017	Redo mitral valve replacement	PE, FFP, Factor VIII concentrate	Not reported	The patient was Factor Ⅴ and Ⅷ deficiency.
Girolami A, 2008	Exploratory laparotomy and left oophorectomy	Whole blood, FFP	Not reported	
Koduri PR, 2016	Laparoscopic assisted vaginal hysterectomy and bilateral salpingo-oophorectomy	RBC, FFP	Not reported	
Sallah AS, 1996	CABG	DDAVP, FFP, PE, recombinant factor VIII concentrate	Not reported	The patient was Factor Ⅴ and VIII deficiency. The patient developed pulmonary edema.
Coppola A, 2010	Caesarean section	rFⅦa, leukodepleted FFP from a sibling	Not reported	The patient had a history of anaphylactic reactions to FFP.
Baron BW, 2001	Case 1: Endoscopy Case 2: Elective abdominal hysterectomy and bilateral salpingo-oophorectomy	Case 1: FFP, PE, platelet packs Case 2: S/DP, PE	Not reported	
Bartlett JA, 1985	Exodontia	Cryoprecipitate, FFP	Not reported	The patient was Factor Ⅴ and VIII deficiency.
Lee WS, 2001	Ventriculoperitoneal shunt	FFP, platelet concentrates, exchange transfusion, Autoplex	Not reported	
Melliger EJ, 1971	Cholecystectomy	Fresh plasma	Not reported	
Tsuda H, 1990	Palatoplasty and tooth extraction	Frozen plasma, RBC	Not reported	On the operation day, 300 ml of blood were drawn from the patient, and he received the same volume of the frozen plasma. The blood obtained from the patient was centrifuged and the red blood cells were returned to him both during and after the operation.
Rideau C, 2010	Total knee arthroplasty	FFP, blood salvage	Not reported	
Mouton C, 2019	Transcatheter aortic valve implantation	Platelets	Not reported	It was determined that a massive infusion of FFP would risk circulatory overload, so platelets were transfused instead.
Yotsumoto G, 2005	Off-pump CABG	FFP, autologous donation	Not reported	
Schultz SC, 1997	Emergent pericardiocentesis followed by complete pericardiectomy	RBC, FFP	Not reported	
Mapp SJ, 2011	Mitral valve repair	FFP, platerets	Reported	
Chava SP, 2006	Splenectomy and proximal lieno-renal shunt	FFP	Reported	
Mathias M, 2013	Case 1: Insertion of a Hickman line, Repair of the Tetralogy of Fallot, Ventricular septal defect closure, Atrial septal defect closure Case 2: Conversion of Hickman line to a port-a-cath Case 3: craniotomy and evacuation of haematoma	Case 1: MB FFP, SD FFP, FFP, platelets, rFⅦa Case 2: MB FFP, SD FFP, platelets Case 3: MB FFP, SD FFP, platelets, rFⅦa	Not reported	
Fu YX, 1996	Emergency craniotomy for evacuation of the subdural hematoma	FFP, PC, combination of PE and chemotherapy	Not reported	The patient deteriorated after initial treatment with FFP and platelet transfusions. He was subsequently treated with a combination of plasma exchange and chemotherapy, which led to complete recovery.
Bauduer F, 2004	Prostatectomy	FFP, desmopressin	Not reported	The patient was Factor Ⅴ and VIII deficiency.
Ichikawa H, 2000	Total gastrectomy	Fresh blood, FFP	Not reported	
Tanis BC, 1998	Excision of a pseudotumour	FFP	Not reported	
Winckelmann G, 1968	Tooth extraction	exchange transfusion, FFP	Not reported	
Takeuchi M, 1984	Tooth extraction	Fresh plasma	Not reported	The patient was Factor Ⅴ and VIII deficiency.
Boinot C, 2004	Knee arthroscopy	VI FFP	Not reported	
KATO Y, 1963	Left bronchotomy	Fresh blood	Not reported	The patient developed pulmonary edema.
Howard C, 2017	Repeat mitral valve replacement	plasma exchange, intravenous factor replacement, and platelet transfusion	Not reported	
Nakashima K, 2021	Mitral valve repair	FFP, PC	Not reported	
Yousef S, 2019	CABG	FFP, PC	Reported	

In other efforts to prevent volume overload, there were also reports of the transfusion of PC instead of FFP in anticipation of the effect of factor Ⅴ in platelets (Table 2: No. 13). Several reports have indicated that platelet function plays an important role in the hemostatic mechanism in patients with FⅤ deficiency [1,11,12]. FⅤ is synthesized mainly in the liver and released in the bloodstream; however, it is also stored in platelet α-granules and megakaryocytes, with approximately 80% of FⅤ being present in plasma and the remaining 20% being present in platelet α-granules [1,12]. FV-deficient patients have low plasma levels of the anticoagulant protein tissue factor pathway inhibitor, which considerably reduces the FV requirement for thrombin generation [1], so it is thought that FⅤ in platelet α-granules alone can produce sufficient amounts of thrombin for hemostasis [1,11,12]. Some reports suggest that the severity of bleeding depends on the FⅤ levels in platelets [2]. Thus, coagulation factors and platelets play an important role in the perioperative management of patients with FⅤ deficiency. In this regard, the decision to transfuse platelet concentrates preoperatively and the use of the TEG to evaluate the hemostatic mechanism of the coagulation factors and platelets combined were useful in the present case.

Finally, the cause of platelet dysfunction in this patient is unknown; however, since it has been reported that patients undergoing maintenance hemodialysis are generally prone to platelet dysfunction [13], long-term hemodialysis could be the cause of platelet function decline in this patient.

## Conclusions

This case report demonstrates the potential for perioperative blood transfusion management in patients with FⅤ deficiency undergoing cardiac surgery by determining whether plasma, cryoprecipitate, or platelet replacement should be performed using TEG.

In this study, perioperative blood transfusion management of CABG in a patient with FⅤ deficiency complicated by acquired platelet function disorders was safely performed using TEG. TEG may be actively utilized to assist in appropriate blood transfusions.
